# “The facilitator is not a bystander”: exploring the perspectives of interdisciplinary experts on trauma research

**DOI:** 10.3389/fpsyg.2023.1225789

**Published:** 2023-08-23

**Authors:** Sarita Hira, Madeleine Sheppard-Perkins, Francine E. Darroch

**Affiliations:** Department of Health Sciences, Carleton University, Ottawa, ON, Canada

**Keywords:** post-traumatic stress disorder (PTSD), trauma-and violence-informed care, trauma-informed practice, physical activity, qualitative research

## Abstract

**Objective:**

This study investigates the concepts, knowledge, and guiding principles that inform the practice of professionals researching trauma or working directly with individuals who have lived and living experiences of trauma. These aspects are explored with the aim of identifying current practices and potential gaps which may contribute to more trauma-informed biomarker-based research approaches.

**Method:**

The perspectives of experts were explored through semi-structured interviews with seven participants; these individuals represented trauma research, clinical practice, and trauma-informed physical activity domains.

**Results:**

A thematic analysis of the collected data revealed three focal areas highlighted by participants from all disciplines: “If I want to know trauma in the body of a person I need to know the person’s language” which related to experiences of discussing trauma with clients; “What all people need is a safe place” relayed the importance of safety for participants working with the trauma expert; and “the facilitator is not a bystander” framing trauma-related work as a collaborative process between participants and their care providers.

**Conclusion:**

Evidence of formal implementation of trauma-informed practices within research settings is lacking. This gap is identified within background literature, while the importance of implementing these practices is emphasized by the participants of this study. This presents an opportunity to apply the insights of the interviewed experts toward advancing trauma research methodologies. Adapting biomarker-based research methodologies to fit a trauma- and violence-informed model may have benefits for the quality of participant experiences, research data, and knowledge of effective interventions.

## Introduction

Evidence of burden that trauma and post-traumatic stress disorder (PTSD) places on those affected is well established; this burden presents itself in terms of psychosocial impacts as well as socioeconomic effects. In the United States alone, it was estimated that the total excess economic burden of PTSD was 232.2 billion in 2018 (Davis et al., 2022). On an individual level, PTSD has been associated with worse physical and mental health functioning, increased disability including development of chronic pain or physical conditions, and disrupted day-to-day-functioning (Goldberg et al., 2014; Atwoli et al., 2015; Herrera-Escobar et al., 2021).

Growing recognition of the effects of PTSD has contributed to substantial efforts to study the accuracy of various biological measures of trauma as a means of better understanding this pathophysiology. However, there is a dearth of research centered around the experiences of participants with biomarker measures, particularly in a trauma research context. Biomarkers like neuropeptide-Y (NPY) and cortisol have been well-established as having significant associations to PTSD; in the case of both biomarkers, these relationships are compelling enough that they have been used in interventional studies using PTSD animal models and human participants (Aerni et al., 2004; Schemletzer et al., 2016; Speer et al., 2019; Nwofakor et al., 2020). These are far from the only biomarkers being used to investigate the pathophysiology of PTSD and trauma exposure, with other measures including inflammatory marker IL-6 and a range of steroid compounds such as allopregnanolone + pregnanolone combinations, testosterone, and DHEA-sulfate (DHEAS) (Kim et al., 2020). Clearly, there is extensive literature about the potential validity and significance of a wide variety of biomarkers that may relate to trauma and PTSD, yet investigations of the impacts of biological measures that may be invasive or uncomfortable for participants appears to be limited.

In the case of participants impacted by trauma and/or PTSD, qualitative evaluation of these experiences may be particularly relevant as demonstrated in the next section by adjacent research investigating the possible re-traumatization that can be associated with physical exams and other healthcare procedures. The nature of the procedures necessary to collect certain biomarkers may range from minimally invasive, as in the case of salivary cortisol collections, to more intense protocols such as blood draws or even lumbar punctures, which are used when measuring biomarkers like NPY (Speer et al., 2019; Kim et al., 2020). Collecting data with a high level of validity and accuracy may mean that participants undergo extensive screening or regulatory procedures with physical exams and vitals collection, laboratory and/or toxicology tests, and even fasting prior to sample collection for lumbar punctures (Kim et al., 2020). While the need for these protocols is clear in terms of ensuring that meaningful information can be obtained from the study, the appropriateness and tolerability of these measures for participants affected by trauma and/or PTSD remains unclear.

## Lived and living experiences of trauma in a healthcare setting

Healthcare settings are sites where individuals are meant to experience healing, but may instead experience trauma. In particular, patients with a history of trauma may be at risk of re-traumatization when re-entering these spaces. Receiving care in these settings frequently involves physical exams and procedures in addition to history-taking by healthcare providers, demonstrating that the dialogue surrounding potential avenues of re-traumatization extends far beyond recall-based approaches whether in clinical practice or research (Watson, 2016; Fleishman et al., 2019; Schippert et al., 2021). Aforementioned, there are significant impacts of trauma and PTSD on physical as well as mental health. A robust body of evidence reveals a heightened vulnerability to serious conditions, such as chronic cardiovascular disease, liver disease, depression, substance use and sexually transmitted diseases, amongst those with past or ongoing exposures to trauma (Liebschutz et al., 2007); thus, primary healthcare settings are highly populated with individuals affected by trauma (Cronholm et al., 2015; Fleishman et al., 2019).

Indeed, examples of potentially re-traumatizing procedures in the medical system are diverse, ranging from environmental or spatial triggers (i.e., small, non-private exam rooms), physical triggers (i.e., undergoing a medical procedure) or interpersonal triggers (i.e., the gender of your healthcare provider) (Center for Substance Abuse Treatment, 2014). Re-traumatization may occur during intimate healthcare procedures or seemingly non-invasive stimuli, such as the weight of an x-ray apron or the tightness of a blood pressure cuff (Reeves, 2015). This somatization is particularly relevant for survivors of physical and sexual violence, for whom traumatic memories are tied to physical sensations and who can be triggered by physical exams, particularly those that involve sites of abuse such as genitals and breasts (Roberts et al., 1999; Leeners et al., 2007). In addition to the physical components of the healthcare experience that may trigger traumatic associations, the interpersonal dynamics of physical exams can imitate those of abuse or violence, such as being told to relax and/or feeling trapped or restrained (Reeves, 2015). Review of the literature reveals the precarious nature of physical exams, which have the potential to foster trust and sentiments of care between the client and medical professional, or as noted, act as a catalyst for re-traumatization or feelings of shame, vulnerability, discomfort and/or distress (Center for Substance Abuse Treatment, 2014; Schulman and Menschner, 2018; Elisseou et al., 2019).

While much of this research focuses on physical exams and sexual violence, it is critical to note key commonalities that should be considered in less routine and/or researched medical procedures. For example, feelings of restraint, physicality, invasiveness and immobilization are overlapping “triggering” characteristics shared with procedures such as neuroimaging, taking vitals, drawing samples, and surgical procedures. Thus, while there is a gap in empirical research on the trauma-sensitivity of practices beyond physical exams, the many ways in which medical procedures can act as a mechanism for re-traumatization is clear.

It should be noted that there has been important work done related to advancing trauma-informed care practices for people affected by trauma and/or PTSD within non-research settings. Current trauma- and violence-informed care (TVIC) literature provides a prime example of how the existing evidence base may be applied to research. TVIC has been defined as, “recognizing that most people affected by systemic inequities and structural violence have experienced, and often continue to experience, varying forms of violence with traumatic impact. Such care consists of respectful, empowerment practices informed by understanding the pervasiveness and effects of trauma and violence” (p.5) (Browne et al., 2015). A TVIC approach relies on four key principles: understanding trauma, violence, and its impacts on people’s lives and behaviors; creating emotionally and physically safe environments for clients and service providers; fostering opportunity for choice, collaboration and connection; and providing a strengths-based and capacity-building approach to support client coping and resilience (Ponic et al., 2016; Arthur et al., 2023). In recent years, there has been momentum in the uptake of TVIC practices in a diversity of settings, such as primary health care (Browne et al., 2015), mental health and social work services (Harris and Fallot, 2001; EQUIP, 2018), school systems (Rodger et al., 2020), homeless social services (Hopper et al., 2010), and physical activity programming (Darroch et al., 2020).

Recommended clinical practices from relevant literature to address the potential of re-traumatization align with the outlined key principles of TVIC and with the previously discussed findings from studies of re-traumatization within healthcare settings. These practices include providing patients with access to a diverse staff, including access to staff of the patient’s preferred gender, as well as providing the patient with choices about how to receive their treatment (Vaughn, 2021; DeMaria et al., 2023). Describing the specific steps to be done during a physical exam and explaining why these steps are being taken has also been highlighted as important “precautionary care” for preventing clinician-induced trauma (DeMaria et al., 2023, p. 6). Given the frequent collaboration of clinicians with researchers on trauma and PTSD-focused studies, it is possible that these trauma-informed approaches are in fact being applied while conducting biological research. Where there remains a gap, however, is in terms of documented trauma-informed approaches specific to procedures used for the collection of biomarkers, for example the collection of blood or cerebrospinal fluid. Additionally, even if practitioners are approaching physical exams and procedures conducted for research studies with a trauma-informed lens, this approach is not mentioned in published descriptions of the relevant methodology. Documenting the use of trauma-informed approaches within research has relevance as a matter of ensuring that this is standard practice, particularly given the potentially re-traumatizing effects of trauma within clinical settings as discussed in the previous section.

## Lived and living experiences of trauma in a research setting

Over the past two decades, there has been an ongoing discourse surrounding the ethical considerations and subsequent consequences of conducting research with individuals who have lived and/or living experience of trauma such as interpersonal and sexual violence (Newman and Kaloupek, 2004; Becker-Blease and Freyd, 2006; Black and Black, 2007). The nuanced and complex nature of this debate is reflected in the literature, with converse findings on the relative risks and benefits of trauma-related questioning in research (Jaffe et al., 2015). While institutional research boards (IRBs) continue to express major concern over study protocols that include questioning participants about past traumas (Yeater and Miller, 2014), the majority of research suggests that these notions of harm are often overemphasized, in turn perpetuating a societal hesitancy amongst trauma populations to disclose their experiences (Griffin et al., 2003; Newman and Kaloupek, 2004; Becker-Blease and Freyd, 2006). Several recent reviews support this body of work, finding that the risk–benefit ratio for research related to experiences of trauma were not unfavourable (McClinton Appollis et al., 2015), and that participants were significantly more likely to report benefits than harm from their research participation experience, regardless of gender and type of trauma exposure (Hebenstreit and DePrince, 2012; Jaffe et al., 2015; McClinton Appollis et al., 2017). The current evidence base provides important insights into the benefits of trauma-focused research, but it does so by focusing mostly on the psychosocial aspects of this area of study. The risk–benefit ratio for participants engaged in biomarker-based research remains uncertain. Thus, although it is crucial to ensure that studies investigating the psychosocial elements of trauma are trauma-informed, this article focuses on the biological research setting.

## Bridging the gap

The majority of exploratory work on the cost–benefit ratio of conducting research with trauma populations focuses on self-report and semi-structured methods (i.e., “talking through” their traumatic experiences) (Griffin et al., 2003; Becker-Blease and Freyd, 2006; Black and Black, 2007); review of the available literature reveals a dearth of research on the appropriateness, feasibility and potential for re-traumatization when using biological trauma measures in a research context. Biomedical procedures, such as physical exams, blood or saliva sampling and neuroimaging, are commonly used in research settings to glean a better understanding of the biomarkers of trauma (Hull, 2002; Bartholomeusz et al., 2017; Pan et al., 2018; Short et al., 2019; Pan et al., 2020). As such, there remains a major gap in understanding the best practices for a major area of research within the discipline of trauma-focused work.

To address this gap, our research takes an interdisciplinary approach that engages trauma experts in research, clinical practice, and trauma-informed practices. By interviewing experts across multiple areas of trauma-related work, this study seeks to reveal the priorities and insights of different disciplines focused on trauma, and demonstrate the ways in which trauma researchers and practitioners consider the lived experiences and practical needs of people impacted by trauma. The multi-faceted understanding of trauma promoted by this study may provide much-needed insights into strategies that can improve the quality of participants’ experiences in the delivery and application of trauma-focused research that employs biomarker measures.

## Methods

This research was approved by the Research Ethics Board at (educational institution) (Clearance #112348). The recruitment of potential interviewees also reflects the importance of cross-sectoral insights and expertise in this study, whereby participants included researchers, care providers, and practitioners working with populations impacted by trauma. The inclusion of both researcher and practitioner/care provider perspectives was critical for interdisciplinary insights into a trauma-informed framework for biomarker research. While researchers bring scientific expertise, practitioners and care providers offer real-world applicability and insights from direct interactions with trauma-impacted individuals, ensuring the recommendations are relevant and feasible in clinical settings. This holistic approach considers the multifaceted aspects of trauma and prioritizes patient-centered care, leading to tailored and culturally sensitive guidelines that accommodate diverse populations. Ethical considerations are also enhanced, as practitioners and care providers contribute valuable input on potential risks and benefits, promoting ethical research practices. Moreover, the collaboration fosters knowledge sharing, allowing for innovative solutions and improved healthcare practices to ultimately benefit trauma-impacted populations.

Interviewees were selected using a combination of purposive sampling and snowball sampling. Applying these methodologies, potential participants were sent an email invitation based on their status as a professional working in trauma-related research or practice, aligning with the purposive sampling approach of selection based on expertise in an area (Palinkas et al., 2015). At the end of each interview, participants were asked to share researcher contact information with colleagues whose work aligned with the goals of this project, representing the snowball sampling component of the recruitment methodology (Parker et al., 2019).

The semi-structured interviews (*n* = 7) with experts from Canada and the US were conducted by the first author. The interview questions in this guide were created to understand the experiences of experts in providing trauma-focused care or researching trauma. This guide included questions focused on obstacles to providing care and how biological knowledge of trauma and/or PTSD informed the work of participants, such as: What are the key components or concepts when it comes to the biology of PTSD, and how does that inform your work (if at all)? and What is the biggest obstacle you have encountered when it comes to providing care/doing research with trauma exposure and/or PTSD?

Interviewees consented to participating in the study and indicated their preference of having their insights attributed to them or having their responses anonymized. The interviews were conducted remotely via Zoom Video Communications Software between October 2020–December 2020 and lasted between 20 to 60 min. These interviews were digitally recorded and transcribed. Following the transcription of interview data, participants received copies of their transcripts for verification purposes. No changes to any of the transcripts were requested. The transcripts were entered into the qualitative data analysis software program, NVivo^™^, for storage and coding.

The seven participants represented three broad disciplines within trauma research and practice. Two interviewees were neuroscience researchers involved in lab-based and/or clinical research, three interviewees were trauma-informed practitioners whose work focused on trauma-informed physical activity, and two interviewees were physicians who had experience working with patients affected by complex trauma. One of these physicians was involved in clinical research as well as practice. The conditions of COVID-19 created challenges to connecting with additional trauma experts; due to this context as well as the diversity of participants’ areas of expertise, data saturation was not achieved. Potential interviewees continued to be invited to participate throughout the interview period, with interviews continuing until no further responses were received from potential participants. The background information of the seven interview participants can be seen in Table 1, which has been provided as a supplemental material.

**Table 1:** Considerations for biomarker-based research involving trauma-affected participants.

Trauma- and violence-informed care principle	Recommendation(s)
Trauma awareness: understanding trauma, violence, and its impacts on people’s lives and behaviour	Ensure trauma and violence awareness is built into the culture of the research organization or group Pay attention to participants’ non-verbal cues, check in with them regularly and avoid sudden movements during collection.
Emphasis on safety: creating emotionally and physically safe environments for clients and service providers	Collaborate with trusted individuals or organizations within the target community to assess the appropriateness of the selected biomeasure for community members and suggest any strategies that would improve participant experiences. Coordinate with trusted individuals or organizations within the target community to develop recruitment strategies that are deemed appropriate by the community and that will not exclude participants who may not have access to resources necessary for explicit PTSD diagnosis (ex. Use self-disclosed experiences of trauma as an inclusion criterion and incorporate a PTSD scale as a study measure). Provide situation-based and culturally-specific training based on input from the target community to improve emotional, physical, and cultural safety of research practices. Provide clear explanations of research procedures and what participants can expect to sense or experience during biomarker collection (e.g., slight discomfort or momentary pain associated with blood draw).
Choice and collaboration: fostering opportunity for choice, collaboration and connection	Actively listen to participant questions and/or concerns. Give participants options about their participation – allow participants to move to a more private location, to sit or stand, and to take breaks if desired.^a^ Let participants have a support person present during biomarker collection, such as a family member, friend, or community member who will increase their comfort.
Strengths-based and capacity building: provide a strengths-based and capacity-building approach to support client coping and resilience	Work in coordination with trusted community members, experts, and/or organizations during the design and execution of studies to ensure that new research projects account for the areas of knowledge and/or intervention type(s) that are valued by members of the target community.

a The authors recognize that the stated examples may not be possible to implement without disrupting measurement accuracy for some biomarker collection procedures. These recommendations present only a limited number of potential applications of the findings of this study, and researchers seeking to incorporate a trauma- and violence-informed approach in their research should be encouraged to adapt these recommendations to fit the nature of their work.

Braun and Clarke’s six-step thematic analysis process was used in this study. In the first phase, the authors immersed themselves in the data by listening to recordings and reading and re-reading transcripts. In the second phase, the authors generated preliminary codes and collated the data into meaningful groups. The third phase involved breaking down the preliminary codes into overarching themes and in the fourth stage the authors met and discussed the themes and sub-themes in the data, ensuring relevance to extracted data. In the fifth step, the themes were defined and named, and a more detailed analysis was developed. Finally, during the sixth phase, all of the data for analysis was compiled to produce this manuscript.

## Results

There were three major themes identified across participants’ responses. The first theme, *“If I want to know trauma in the body of a person I need to know the person’s language”*, focused on experiences of disclosure and discussion of trauma with clients; the second theme, *“what all people need is a safe place”*, centered around the importance of safety for those working with the trauma expert; and the final theme, *“the facilitator is not a bystander”*, framed trauma-related work as a collaborative process between participants and experts.

### If I want to know trauma in the body of a person I need to know the person’s language

Interviewees expressed distinct experiences with their clients’ disclosure of trauma history, and more broadly with the language used by those impacted by trauma. David Emerson, a trauma-informed practitioner emphasized the “non-verbal nature of trauma”; accordingly, this expert felt that trauma exposure did not require a diagnosis. Instead, Mr. Emerson felt that his clients required “an insight and then just availability” from him. This framing of the relative responsibilities of client and practitioner align with the experiences of a physician who works with patient populations impacted by complex trauma. As Dr. Adams, the selected pseudonym for this participant, explained,

It’s probably 30–30-30… so there’s a third who will be very explicit about things that have happened, there’s a third who sort of allude to them. And there’s a third who will not really talk about it, but you can kind of get a sense from like the chart you are looking at or the way they have accessed care or their hospitalizations or things like that that there’s a lot of trauma history there.

This participant also discussed the relative rarity of a formal PTSD diagnosis among their patients, explaining that “the majority of patients do not have one [PTSD diagnosis] officially…I can count on one hand how many patients have a true diagnosed, like via psychiatric assessment, PTSD diagnosis”. The clinician explained that they tried to acknowledge these behaviors through a trauma-informed care approach, but that they “often do not go into the details of what we might need to do to officially identify someone (having) PTSD.” This approach was explained as due to a combination of lack of time and capacity as well as concern that “we may not be able to fully support that patient if we start like poking and prodding at parts of their lives and their experiences, that like potentially might re-traumatize them.” These concerns may also be linked with Dr. Adams’ discussion of issues with the quality of care received by patients.

A lot of patients will actually tell us about like traumatizing or re-traumatizing experiences they have had in healthcare. And I think that it – like particularly those of us working in the population, we understand that there’s this challenge in the systematic ways in which healthcare re-traumatizes and re-stigmatizes people. But I do not think we always know how to…overcome that or sometimes how to reshape how we offer care in a way that is better for people.

Interviewed researchers expressed complementary experiences to their clinical and practitioner counterparts with the disclosure of trauma by participants. In reference to a study with participants who were Somali immigrants, neuroscience researcher Dr. Hymie Anisman, described his experience with collecting data about trauma history and the impact of trauma type on level of disclosure:

during the course of the interviews with the Somali immigrants [we] ended up asking them about sexual trauma they encountered, at which point every last person just stopped talking and they would not continue with the study…As soon as that issue came up, we thought it might be a problem, so we kept it at the end of the interviews and questionnaires. But this was clearly a big deal, but you could not get any answers. So sometimes, your participants are not going to give you the information you need to get.

This discomfort with disclosure was also present in a study this researcher conducted with university students, which was suggested to be attributed in part to the nature of the research context:

They too were often reluctant to go into too much detail, OK, but they were, they were more open. But again, that seemed to be something that they would probably share with their physician, their psychiatrist, or the law enforcers, but not with researchers.

The other interviewed neuroscientist, with the pseudonym Dr. Brown, described clinical research experiences which were done in collaboration with a team of healthcare workers. This study’s inclusion criteria incorporated a validated PTSD measure, meaning that participants had already been diagnosed and as a result seeking disclosure of these experiences was not necessary for the researcher.

Most of the patients that I’ve met, or used in our study, yes. So they had a diagnosis. They already had what’s called the CAPS, Clinician-Administered PTSD Scale, and they had already been categorised as mild, severe, moderate PTSD.

Interviewees expressed diverse perspectives when it came to describing high priority aspects of the biology of trauma. Trauma researchers highlighted the limitations of applying knowledge from animal model research to humans, with Dr. Brown expressing that they were “not fully convinced that our research is answering some questions.” Mark Schneider, a trauma-informed practitioner similarly described limitations centered around the perceived applicability of biological trauma research to his practice. Mr. Schneider reported that he rarely discusses the biology of trauma with his clients due to his desire not “to add another anchor for them [clients] to be able to attach something to….I do not want to blame their response, their reaction, their experience on anything regardless of what it might be.” Instead, this participant described his strategy of adopting the language used by the client.

If I want to know trauma in the body of a person I need to know the person’s language so then I have to learn what, how are they describing it, what their experience is, what are their behaviors around it, what are their perceptions, their original belief system…

Mr. Schneider expressed that in his practice, he focuses on “how this person is functioning in space…their perceptions and understandings and beliefs…” and if biological terms were being used by the client, “how they are using [biological] languages…habitually as a limiting belief system.” This view contrasted with the perspective provided by another trauma-informed practitioner, Mariah Rooney, who expressed that “a lot of us start from a place of listening and lived experience, but also, we want to know what’s happening in people’s bodies, because that will help us be even more, be informed in how we approach the work.”

### What all people need is a safe place

The role of support was described as a key aspect of the relative accessibility and quality of care across disciplines. Dr. Adams described the impact of “masculinity and ways in which particularly urban Indigenous men are, or have historically, been socialized in sort of a colonial context” as a significant barrier to accessing care for their patients. This focus on the impact of the ethnocultural aspects of patient experiences was also discussed by neuroscience researcher Dr. Anisman.

Stigma was discussed as a key component of the possible negative impact of cultural norms:

Where your culture (referring to culture more broadly), for example, may turn on you and say, “You’re weak for showing this PTSD. There’s a weakness in you.” And we see this all the time with people with mental illnesses and PTSD is amongst them. And so, people hide. It’s not just you and I, they just do the same thing. They do not disclose, because they know there’ll be stigma.

Complementing this explicit discussion of stigma, trauma-informed practitioner Mr. Emerson pointed out the role of the framing of trauma and illness more broadly in society.

There’s a dichotomy in traditional treatment where there’s a sick person or there’s – you know; however, you want to break it down, there’s somebody who’s like not well and somebody who’s well, you know, in general. And I think complex trauma is so potent in terms of exposing the problems with that way of thinking. None of us are well.

The role of isolation and support was highlighted by a trauma researcher and a practitioner. Dr. Anisman expressed that “one of the biggest stressors in our research is referred to as unsupportive relations.” Similarly, Mr. Emerson described how “trauma (is) about like separating. You know, it’s really – it’s driven by separating people,” thus emphasizing the role of disconnection in the impact of trauma. Dr. Anisman expanded on this focus on support by expressing that “What all people need is a safe place…I do not necessarily mean a physical place…but a safe place in your head, so you can say, ‘It’s over. It’s gone. I can now move on’.” The researcher also discussed the interaction between the physical and psychosocial aspects of trauma, explaining that “if you have a social stressor present, the effects of a physical stressor would be increased. And vice versa, if there’s a physical stressor present, the effects of social stressor would be increased.”

Safety and trust were similarly highlighted by Dr. Adams, who described the potential challenges of the clinical environment for patients who have experienced trauma:

I have a few patients who like never want their name called out in a waiting room or a waiting area because they have had…experiences of like ex-partners stalking them or people looking for them and they sort of want to remain anonymous but they really do trust us.

This need to prioritize safety and comfort was echoed by trauma-informed practitioners and trauma researchers alike. Mr. Schneider spoke about the “inability to trust self, others and environment” as one of the key factors underlying trauma responses. This participant further highlighted the importance of trust while discussing his experiences of working in trauma-informed weight lifting. Trauma-informed weight lifting is described on the program website as “An embodied practice and intervention that…seeks to transform weight lifting in an effort to both promote and facilitate healing for trauma-impacted individuals and groups” (Trauma Informed Weight lifting, 2021). The practitioner explained that in this case, where there is a concrete and external stressor in the form of the weight, trust is crucial.

You have to either trust yourself to be able to go down and put the effort in to come up or the level of trust you need to be able to have to your environment so that if you do come down and you cannot overcome it that you can get out of it. So it’s both the level of trust that you need to succeed and the level of trust you need to have of the potential failure.

Interviewees described experiences of successful outcomes with participants when efforts were made to address these challenges of trust. Neuroscience researcher Dr. Brown described their experience with participants in a study focused on biological measures of trauma as follows:

People were so, so involved and very – no one complained. I mean, I’ve never seen people who are this motivated… I was just amazed with their commitment to the study, their enthusiasm.

The researcher attributed this observed level of commitment and enthusiasm to the positive relationships between participants and the clinical research team. Ms. Rooney similarly expressed the importance of relationships in her work as a trauma-informed weight lifting practitioner. She explained that some of her knowledge of what would be effective for clients came from hearing “a lot of trauma stories and histories from different (weight) lifters in the community.” The quality of relationships within the clinical or research environment appears to be a critical component of the perceived safety of that space, as participants explored in the final theme.

### The facilitator is not a bystander

Interviewees provided further insights into the nature of successful relationships between themselves and the client, patient, or participant. Dr. Ruth Lanius, who is involved in clinical research and practice, described the importance of investing time to build a relationship with patients prior to their participation in a study.

What’s critical is that, you know, the individuals who are there when the patient is scanned and who will have a relationship with the patient before they come in, that they are really well-schooled and that they are very competent clinically and are able to make the participant feel as safe as possible.

Mr. Emerson similarly discussed a relationship-oriented approach when describing the overarching ideals of trauma-informed practice, where there are “five elements that are always at play and one of them is that the facilitator is not a bystander.” He described how according to this approach, the care provider engages in the practice along with the client:

we are approaching these yoga forms together. We’re each having our own real experience with this thing, you know, and we are not interfering with the other person’s experience, but we are sort of sharing the space together.

This practitioner expressed that in this type of practice, participants make conscious choices about their bodies and their movements.

It’s this embodied practice where we each get to be in charge of ourselves, right. There’s no physical assist because we are each in charge of our bodies. Practising noticing what we feel ourselves, making choices about the forms.

From the clinical perspective, Dr. Adams described a similar focus on client perceptions of choice and autonomy:

I really try to let patients decide and patients who – patients will often come forward with either things that have helped them in the past…I’ll often try to elicit from patients what they think would work for them and then my practice tends to be trying to offer patients both like pharmaceutical but also the other types of tools and experiences that a lot of people find helpful.

When measuring the relative progress of a client or the effectiveness of a treatment, interviewees described clinical and trauma-informed practices that center the choices and opinions of participants. Mr. Schneider explained that in his work, he recognized that making a choice could be a major sign of progress for a client, regardless of whether that choice was choosing to engage in an approach or not.

Being able to say yes, I still want to go through with this or more importantly for most people, I think, is being able to say no, I actually do not want this. So that’s, to me in a long term, if I can, if a client is calmly and confidently can say no to me, that is a major progress especially if because I have my approaches, but I do not particularly express opinions on many things. I do my best to create as blank of a slate as I can so that whatever they say it becomes amplified back at them.

Similar to Mr. Schneider’s approach of creating “as blank of a slate” as possible for the client, Dr. Lanius described the inclusion of regular consultations with patients as a part of her practice:

So I think…doing these approaches and then checking in with the patient and saying, you know, “Does this make sense to you, does this feel right or not right” and sort of maybe evaluating day by day.

The focus on choice and collaboration described by clinical and trauma-informed practitioners was not reflected in interviews with researchers, though it should be noted that interviewees were not explicitly asked about these elements. While there were some discussions related to ethics committees, these discussions centered around ethics committee concerns about trauma history questionnaires potentially triggering participants. The experiences of participants were described by Dr. Anisman, yet these descriptions were in relation to the impacts of study participants’ stress on cortisol, the stress hormone and biological measure in focus for the study:

when they come into the lab, they are often feeling anxious, OK. They know why they are there. And even if they do not, their cortisol levels are here. Over that half hour, it declines, OK. And if you were to do nothing to them, they can control subjects, it declines even more. So, when they are coming in, they are not at a real baseline, they are at some stress level.

In a discussion about the comfort of participants with the collection of biological measures, Dr. Anisman also discussed the benefits of providing a private space for participants to provide saliva, which is needed for cortisol measurement.

Most are OK. There’s different ways of doing it. Some are a little bit shy about spit, OK. But if you – if they are in an area where nobody else is looking at them, OK, or if you have them shielded, like a curtain or something like this, then we have never found it to be a problem, OK. Well, I should not say never. We may have had one or two people (who had problems with saliva collection).

Trauma-informed practitioners presented mixed views on the benefits of biomarker-based studies. Mr. Emerson described his perspective of these biological measures as being potentially disruptive due to the requirement for participants to remain immobile. The practitioner linked his discomfort with biological measures to an assessment of the language used by people with lived experiences of trauma, pointing out that biomarker research may have limited usefulness for these individuals.

People talk about that disconnection between their mind and their body that yoga has language for and neuroscience has language for now with interoceptive theories. So people themselves, they are not saying “I have too much cortisol in my system” anymore. You know, it’s more about what’s driving me. It’s so difficult is I cannot – my body and my brain are disconnected.

While discussing the potential usefulness of biological trauma research, Ms. Rooney described the potential benefits of a community-based and participatory approach.

I think that we have to be trauma informed in how we approach research…I think part of being trauma informed is centering the voices of those who are impacted. And so, bringing people in to be a part of research conceptualization, design, methodology, implementation, analysis, all of it, is really important…I think particularly when you think about who the participants are in trauma-based research, they are disproportionately folks who are marginalized and oppressed. And so, how can we approach research in ways that not only centre their voices but also think about [taking] a reparative approach so that we are not – so that folks in positions of power are not benefiting further, right, from the traumatization of oppressed and marginalized folks.

These results collectively show the value of trauma-related work that engages the client, participant, or patient as a partner. Trauma experts across disciplines identified the benefits of addressing trauma in a way that meets the desires and needs of the participant and focuses on relationship-building to improve perceptions of safety. Interviewees also shared their positive experiences with approaches that prioritize collaboration between trauma experts as well as between experts and participants.

## Discussion

Researchers, physicians, and practitioners alike described their attentiveness to the way participants spoke about their experiences of trauma, if they chose to speak about these experiences at all. The interviewed physicians and trauma-informed practitioners revealed that formal PTSD diagnosis and explicit trauma disclosure tend to be infrequent. If the ultimate goal is for trauma-focused research to be translated to these types of practice settings, this may be an important consideration for researchers during study design. These findings may also have implications for the use of validated scales in trauma-focused studies. While the standardization provided by scales such as the Clinician-Administered PTSD Scale (CAPS), a 30-item structured interview that is commonly used to assess PTSD status, is certainly a benefit, it may be important for researchers to consider whether the scale uses phrasing that reflects the language used by the target community when describing experiences with trauma (Weathers et al., 2013).

The existing literature indicates that there may be benefits for participants who disclose their experiences of trauma (Center for Substance Abuse Treatment, 2014; Schulman and Menschner, 2018; Elisseou et al., 2019). Yet based on the findings of this work, study designs that incorporate PTSD diagnosis or trauma disclosure as inclusion criteria may create conditions that do not align with clinical and trauma-informed practice. Even if these research studies do lead to significant findings, these results may not be easily applied to patients who do not choose to disclose their experiences. For interventional studies, the use of trauma disclosure as an inclusion criterion could be a limiting factor to clinical translation of the intervention. In this case, the accessibility of the intervention could be limited to those who are willing and able to explicitly discuss their experiences with their care provider.

Safety and perceptions of support, which were highlighted by experts from all disciplines, may influence the comfort of participants with disclosing their lived experiences of trauma. The interviewed experts described the necessity of a participant’s ability to trust their environment based on the impact of this element on psychosocial and physical well-being. Investing time into relationships with participants appears to be a key strategy among the interviewed experts; some interviewees built these relationships directly with the communities they served, while others collaborated with experts who had already built relationships with the participants.

Cultural safety, which has been defined within the context of trauma- and violence-informed care (TVIC) as an approach that aims to “explicitly address inequitable power relations, institutionalized and interpersonal racism and other forms of discrimination, and the ongoing impacts of historical injustices on health and health care” was not explicitly addressed by participants in this study (Browne et al., 2015). However, some interviewed experts did implicitly recognize the importance of cultural safety, for example by acknowledging cultural norms as a factor in some research participants’ decisions not to disclose certain types of trauma. In discussing biomarker-based studies, one practitioner suggested that researchers focus on recognizing the intersectional marginalizing factors that may impact participants and centering the voices of these communities. Taking into account cultural understandings and approaches to trauma may be an important factor in the quality of participant experiences, and study designs that implement culturally specific approaches as well as acknowledging systemic inequities and structural violence may improve the experiences of certain communities with research (Browne et al., 2015; Government of Canada, 2018). In using this more specific and sensitive type of approach, researchers may further support the construction of a safe research environment.

The role of clinical competence and training was also described as a potential contributor to trust between trauma experts and patients. Trauma-informed practitioners focused on perceptions of autonomy among clients as well as describing an approach that positions the trauma expert and the client as collaborators. This framing appears to be a way to create a shared experience during the trauma-focused treatment, which may oppose perceptions of power dynamics within the therapeutic relationship. This approach may have value in a research setting, particularly for clinical research, due to the negative impact that clinical procedures can have on a patient if interpersonal dynamics contribute to feelings of being trapped or restrained and thus imitate experiences of trauma (Reeves, 2015).

The current evidence indicates that people who are affected by trauma can and do have positive experiences with research (Hebenstreit and DePrince, 2012; Jaffe et al., 2015; McClinton Appollis et al., 2017). The analyzed data suggests that research approaches which seek to incorporate trauma-focused language that reflects the language of participants may improve the generalizability of findings and build trust between participants and researchers. Research approaches that acknowledge the intersectional identities of people with experiences of trauma, which may include distinct cultural identities, and recognize the power dynamics inherent in a research setting may foster greater perceptions of choice and autonomy among participants, a key theme identified in the results of this work. Collectively, the results of this work demonstrate that researchers can take concrete steps to ensure high-quality experience for participants as well as high quality biomarker-based studies.

## Recommendations

The recognition of the importance of trauma- and violence-informed practice is well-established, yet the formal integration of these principles into sensitive trauma research settings are seemingly underdeveloped. Specific training modules for biomarker-based research, which remain nonexistent or underdeveloped, should attend to the key tenets of TVIC, relying on an iterative and participatory approach with members of the community (i.e., past trauma research participants), trauma practitioners and trauma researchers all playing a role. More specific recommendations of how this type of approach may be applied to biomarker-based research settings are provided in Table 1.

Implementing these types of evidence-based training modules for researchers has the potential to act as a feasible tool for IRB boards during the ethics approval process. Further, while ethics reviews may attend to more traditional considerations for trauma research (i.e., types of questions being asked, participant recruitment protocols, etc.), incorporating other key aspects of TVIC that consider complex and ongoing experiences of trauma and marginalization will augment the researcher’s awareness of providing safe, choice-based research spaces.

## Conclusion and future directions

The findings of this study, combined with the robust body of literature on cost–benefit ratios in talk-based research methods, demonstrate the potential benefits of the implementation of TVIC into research spaces. Through the intentional design of studies that are safe, collaborative and value the lived experiences of those impacted by trauma, researchers may be better equipped to produce scientific work that can be translated into effective interventions for a diverse range of trauma-affected communities. Though this research provided critical insight into the trauma-related research and practice experiences from a provider/researcher’s perspective, next steps should prioritize participant-centred experience. Due to the challenges of COVID-19, this study was not able to include the perspectives of research participants. There is also a significant amount of diversity within trauma research, clinical practice, and trauma-informed practice that was not fully captured in these results. Future studies should include perspectives from a more diverse range of participants, in terms of area of work, study or participation as well as demographic and geographic diversity to better capture the broad range of populations impacted by trauma and/or PTSD. Future work should also incorporate qualitative methods which may help develop a better understanding of participant experiences with trauma research.

## Data availability statement

The raw data supporting the conclusions of this article will be made available by the authors, without undue reservation.

## Ethics statement

The studies involving humans were approved by Carleton University Research Ethics Board (clearance # 112348). The studies were conducted in accordance with the local legislation and institutional requirements. The participants provided their written informed consent to participate in this study.

## Author contributions

SH led this research and conceptualized this paper as part of her MSc. MS-P contributed to the conceptualization and writing of this paper. FD supervised this research and supported all aspects of research development, conceptualization, and preparation of this paper. All authors contributed to the article and approved the submitted version.

## Funding

This manuscript draws on research supported by the Social Sciences and Humanities Research Council (grant # 435-2020-0757) held by FD. Written informed consent was obtained from the individual(s) for the publication of any potentially identifiable images or data included in this article.

## Conflict of interest

The authors declare that the research was conducted in the absence of any commercial or financial relationships that could be construed as a potential conflict of interest.

## Publisher’s note

All claims expressed in this article are solely those of the authors and do not necessarily represent those of their affiliated organizations, or those of the publisher, the editors and the reviewers. Any product that may be evaluated in this article, or claim that may be made by its manufacturer, is not guaranteed or endorsed by the publisher.
