# Supplementary material for: “The facilitator is not a bystander”: exploring the perspectives of interdisciplinary experts on trauma research
Source: Front Psychol. 2023 Aug 23;14:1225789. doi: 10.3389/fpsyg.2023.1225789 (PMC10481530; doi:10.3389/fpsyg.2023.1225789)
Supplement: Supplementary file 1 [file Data_Sheet_1.PDF]

Table 1

Interview Participant Background Data

Participant Name or Pseudonym (P)	Area of Expertise	Location
Dr. Hymie Anisman	Neuroscience researcher	Canada
Dr. Brown (P)	Neuroscience researcher	US
Dr. Adams (P)	Physician	Canada
Dr. Ruth Lanius	Physician	Canada
Mark Schneider	Trauma-informed physical activity practitioner	US
David Emerson	Trauma-informed physical activity practitioner	US
Mariah Rooney	Trauma-informed physical activity practitioner	US
