# Supplementary material for: “The facilitator is not a bystander”: exploring the perspectives of interdisciplinary experts on trauma research
Source: Front Psychol. 2023 Aug 23;14:1225789. doi: 10.3389/fpsyg.2023.1225789 (PMC10481530; doi:10.3389/fpsyg.2023.1225789)
Supplement: Supplementary file 2 [file Data_Sheet_2.PDF]

Table 2

Considerations for Biomarker-Based Research Involving Trauma-Affected Participants

Trauma- and Violence-Informed Care Principle	Recommendation(s)
Trauma Awareness: Understanding trauma, violence, and its impacts on people's lives and behaviour	Ensure trauma and violence awareness is built into the culture of the research organization or group<br><br>Pay attention to participants' non-verbal cues, check in with them regularly, and avoid sudden movements during collection.
Emphasis on Safety: Creating emotionally and physically safe environments for clients and service providers	Collaborate with trusted individuals or organizations within the target community to assess the appropriateness of the selected biomeasure for community members and suggest any strategies that would improve participant experiences.<br><br>Provide situation-based and culturally-specific training based on input from the target community to improve emotional, physical, and cultural safety of research practices.<br><br>Provide clear explanations of research procedures and what participants can expect to sense or experience during biomarker collection (e.g. slight discomfort or momentary pain associated with blood draw).
Choice and Collaboration: Fostering opportunity for choice, collaboration and connection	Actively listen to participant questions and/or concerns.<br><br>Give participants options about their participation – allow participants to move to a more private location, to sit or stand, and to take breaks if desired.[a]<br><br>Let participants have a support person present during biomarker collection, such as a family member, friend, or community member who will increase their comfort.
Strengths-based and Capacity Building: Provide a strengths-based and capacity-building approach to support client coping and resilience	Work in coordination with trusted community members, experts, and/or organizations during the design and execution of studies to ensure that new research projects account for the areas of knowledge and/or intervention type(s) that are valued by members of the target community.

Note. [a] The authors recognize that the stated examples may not be possible to implement without disrupting measurement accuracy for some biomarker collection procedures. These recommendations present only a limited number of potential applications of the findings of this study, and researchers seeking to incorporate a trauma- and violence-informed approach in their research should be encouraged to adapt these recommendations to fit the nature of their work.
